# Identification of the MADS-Box Gene Family and Development of Simple Sequence Repeat Markers in *Chimonanthus praecox*

**DOI:** 10.3390/plants14152450

**Published:** 2025-08-07

**Authors:** Huafeng Wu, Bin Liu, Yinzhu Cao, Guanpeng Ma, Xiaowen Zheng, Ximeng Yang, Qianli Dai, Hengxing Zhu, Haoxiang Zhu, Xingrong Song, Shunzhao Sui

**Affiliations:** 1 Chongqing Engineering Research Center for Floriculture, Key Laboratory of Agricultural Biosafety and Green Production of Upper Yangtze River (Ministry of Education), College of Horticulture and Landscape Architecture, Southwest University, Chongqing 400715, China; woo925784127@163.com (H.W.); lb19196321336@126.com (B.L.); yinzhu202108@163.com (Y.C.); guanpengma@163.com (G.M.); wennnn_n@163.com (X.Z.); 2 Institute of Horticulture, Guizhou Academy of Agricultural Sciences, Guiyang 550006, China; 3 Chongqing Key Laboratory of Forest Ecological Restoration and Utilization in the Three Gorges Reservoir Area, Chongqing Academy of Forestry Sciences, Chongqing 400036, China; xm2020@email.swu.edu.cn (X.Y.); daiqianli126@126.com (Q.D.); starring85@sina.com (H.Z.); 4 College of Horticulture and Landscape Architecture, Southwest University, Chongqing 400715, China; zhuhx8910@swu.edu.cn; 5 Garden and Flower Research Center, Horticultural Research Institute of Sichuan Academy of Agricultural Science, Chengdu 610000, China; songxr98@163.com

**Keywords:** MADS-box, *Chimonanthus praecox*, simple sequence repeat markers, flower development

## Abstract

*Chimonanthus praecox*, a traditional ornamental plant in China, is admired for its ability to bloom during the cold winter season and is recognized as an outstanding woody cut flower. MADS-box genes encode transcription factors essential for plant growth and development, with key functions in regulating flowering time and the formation of floral organs. In this study, 74 MADS-box genes (*CpMADS1–CpMADS74*) were identified and mapped across 11 chromosomes, with chromosome 1 harboring the highest number (13 genes) and chromosome 3 the fewest (3 genes). Physicochemical property analysis revealed that all CpMADS proteins are hydrophilic and predominantly nuclear-localized. Phylogenetic analysis classified these genes into Type I and Type II subfamilies, highlighting a clear divergence in domain structure. Eighty simple sequence repeat (SSR) loci were detected, with dinucleotide repeats being the most abundant, and the majority located in Type II MADS genes. From 23 *C. praecox* samples, 10 polymorphic SSR markers were successfully developed and PCR-validated, enabling a cluster analysis that grouped these cultivars into three distinct clusters. This study offers significant insights into the regulation of flowering, floral organ development, genetic linkage map construction, and the application of marker-assisted selection in *C. praecox*.

## 1. Introduction

The MADS gene family, originally identified as homeotic genes, is one of the most extensively studied transcription factor families in plants [1,2]. A defining characteristic is the conserved MADS domain, a DNA-binding region of 58–60 amino acids located at the protein’s N-terminus [3]. Plant MADS-box genes are generally classified into Type I and Type II based on conserved domains and evolutionary relationships [4]. Type I genes have a simpler structure, containing only the MADS domain, and are subdivided into Mα, Mβ, and Mγ groups [5]. In contrast, Type II genes, known as MIKC-type, possess a more complex structure with MADS, intervening (I), K-box domains, and a variable C-terminal region, and are further divided into MIKC* and MIKCC subclasses [6]. MIKCC genes play crucial roles in floral organ development and regulation [7,8].

MADS-box genes regulate key processes in plant growth and development, including flowering time, floral organ identity, fruit ripening, embryogenesis, and vegetative structure formation [9,10,11,12]. They have been extensively characterized in diverse species such as *Arabidopsis thaliana* [5], *Oryza sativa* [13], *Populus trichocarpa* [14], and Ananas comosus [15]. Notably, the ABCDE model of floral organ identity largely involves MADS-box genes, with A-, B-, C-, D-, and E-class genes controlling the development of sepals, petals, stamens, carpels, and ovules, respectively [16,17,18,19]. Except for AP2, which belongs to the AP2/ERF family, all ABCDE model genes are MADS-box members [20]. Despite extensive studies on MADS genes in many plants, their composition and functions in *Chimonanthus praecox* remain unclear. Investigation of the MADS gene family in *C. praecox* characterizes its composition and features, providing a basis for elucidating its roles in flowering regulation and floral organ development.

Recent advances in high-throughput sequencing and bioinformatics have made it possible to identify SSR loci across entire genomes within functional genes, promoting the creation of gene-based molecular markers that are highly specific and practically valuable [21]. Among these, the MADS-box gene family, characterized by its conserved structure and diverse roles in plant development, offers a valuable resource for SSR marker development. For instance, in *Citrus*, researchers have successfully developed 15 SSR markers from MADS-box genes, which demonstrated strong transferability and polymorphism across various Citrus genomes [22]. These studies highlight the potential of functional gene-derived SSR markers not only for analyzing genetic diversity but also for germplasm identification, trait mapping, and marker-assisted selection in plant breeding programs.

Molecular markers have been widely applied in various areas of plant research, including genetic linkage map construction, germplasm identification, heterotic group classification, marker-assisted selection (MAS), gene mapping of important traits, functional gene mining, and quantitative trait loci analysis [23]. Among these, simple sequence repeat (SSR) markers have become particularly popular because of their codominant inheritance, high reproducibility, ease of use, wide genome coverage, and high polymorphism [24]. They have been extensively used in plant taxonomy, new species discovery, classification of variation types, and population genetic structure analyses [25]. In previous research, SSR markers in *Chimonanthus praecox* have been developed from the *CpTPS* gene family [26], and EST-SSR resources have been generated based on transcriptome data [27]. Furthermore, a nine-nucleotide insertion in the second exon of the *FLS* gene in peaches is a key genetic factor responsible for red flower coloration [28]. SSR loci in *CpMYB2* of wintersweet may be associated with anthocyanin biosynthesis [29]. In addition, as mentioned above, SSR markers derived from MADS-box genes in citrus and sweet orange exhibit strong transferability across different genomes [22], suggesting the broad applicability of this strategy in diverse species. In long-lived woody plants like wintersweet, developing molecular markers based on functional genes is particularly important. Due to their long generation times and late trait expression, traditional breeding is inefficient. Molecular markers such as SSRs enable early selection and stable inheritance, facilitating germplasm identification, key trait analysis, and implementation of marker-assisted selection (MAS) breeding strategies, thereby improving breeding efficiency.

*C. praecox* (wintersweet) is a deciduous flowering ornamental species, classified within the Calycanthaceae family and indigenous to China [30,31]. As an important landscape plant, it is widely used in countries such as the United States, Japan, and South Korea [32,33]. In recent years, wintersweet has become an increasingly popular woody cut flower material owing to its unique winter blooming period and graceful floral appearance [34,35,36]. Meanwhile, wintersweet also has medicinal properties such as clearing heat and detoxification [37,38]. As primary ornamental features, the morphology and flowering time of wintersweet flowers are critical for horticultural applications and cultivar improvement [39]. MADS-box transcription factors are key regulators involved in the development of floral organs and the control of flowering time [40]. Previous studies have shown that several MADS genes in wintersweet have important functions in developmental regulation. For instance, transgenic plants overexpressing *CpFUL* (designated *CpMADS12* in this study) exhibited early flowering and abnormal floral organs, such as double carpels and curled sepals [41]. The overexpression of *CpAGL6* (*CpMADS44*) in *Arabidopsis* significantly promotes flowering and causes variation in various floral organs [42]. Similarly, ectopic expression of *CpAP3* (*CpMADS60*) in petunias leads to diverse alterations in floral structures [43]. The heterologous expression of *CpAGL2* (*CpMADS22*) in *Arabidopsis* may be involved in stamen development [44]. *CpFUL* (*CpMADS12*), *CpSEP* (*CpMADS20,21,22*), and *CpAGL6s* (*CpMADS44,47,66*) are involved in dormancy release and bud formation in wintersweet [41].

MADS-box transcription factors act as vital regulators of flowering time and floral organ formation, coordinating crucial developmental processes in diverse plant species [40]. Thus, an in-depth investigation into the MADS-box gene family in *C. praecox* is of great theoretical importance for uncovering the molecular mechanisms that control its flowering and floral organ formation. This study aims to systematically identify the MADS-box gene family members in wintersweet through bioinformatics approaches. The phylogenetic relationships, gene structures, and conserved motifs were thoroughly examined. Moreover, SSR molecular markers derived from MADS genes were developed to evaluate the genetic diversity and population structure among various *C. praecox* cultivars. These results not only help to uncover the genetic basis of floral organ number variation in wintersweet but also provide important candidate gene resources and theoretical references for future functional studies of MADS-box genes, germplasm evaluation, and marker-assisted breeding strategies in *C. praecox*.

## 2. Results

### 2.1. Identification and Physicochemical Characterization of CpMADS Genes

Using HMMER v3.0 software and Pfam domains (PF00319 and PF01486), the *C. praecox* genome was scanned to identify and verify genes with complete MADS domains. A total of 74 MADS genes were identified and named CpMADS1 to CpMADS74 according to their chromosome positions. The physicochemical properties of the 74 CpMADS proteins were systematically analyzed. The amino acid lengths ranged from 54 to 912 residues, with CpMADS32 (Cpra06G00287.1) and CpMADS53 (Cpra08G01360.1) being the longest. Their molecular weights ranged from 6.06 to 99.62 kDa, and their isoelectric points (pI) ranged from 4.20 to 10.29. The instability index values ranged from 30.2 to 88.41, the aliphatic index from 63.29 to 95.96, and the GRAVY (Grand Average of Hydropathicity) values varied between −0.865 and −0.206, suggesting that all CpMADS proteins are hydrophilic. In-silico subcellular localization analysis indicated that all CpMADS proteins localized in the nucleus (Table S1).

### 2.2. Phylogenetic Tree Analysis of the Wintersweet MADS Protein Family

**Figure 1:**
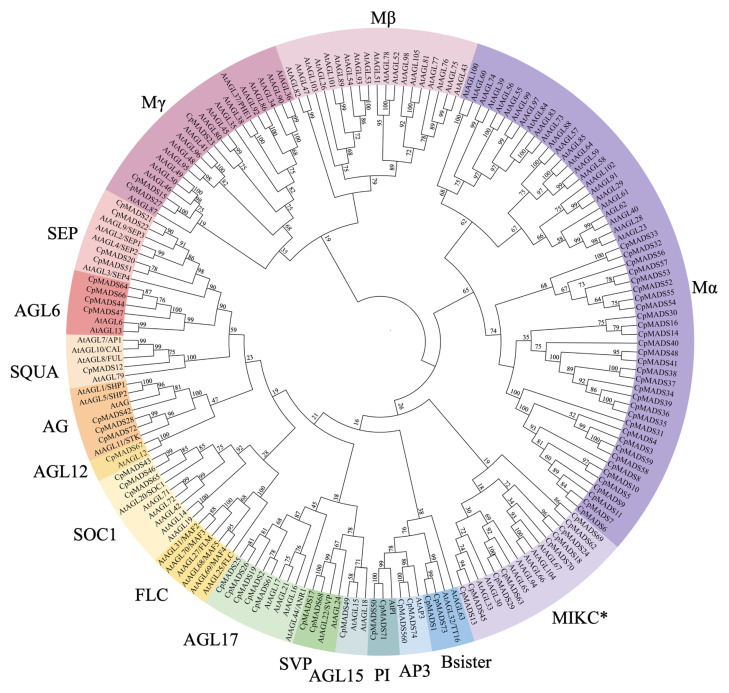
Phylogenetic relationships of MADS in *C. praecox* and *A. thaliana* constructed using the neighbor-joining (NJ) method. Bootstrap analysis was performed with 1000 replicates, and the tree was generated using MEGA X software.

A phylogenetic tree was generated using the neighbor-joining (NJ) method based on the MADS-box proteins from *C. praecox* and *A. thaliana*. The results revealed that among the 74 identified CpMADS proteins, 35 belonged to the Type I group, including 20 members of the Mα subfamily, and 15 members of the Mγ subfamily, with no Mβ subfamily proteins being detected. The Type II group consisted of 39 genes, of which nine belonged to the MIKC* subfamily, while the other 30 MIKCC members were distributed among 13 distinct subfamilies. Except for the *FLC* subfamily, all the other subfamilies were represented in wintersweet. Among them, the AGL17 subfamily contained the highest number of members (five), whereas the SQUA, AGL12, and AGL15 subfamilies each contained only one member (Figure 1).

### 2.3. Analysis of CpMADS Gene Structure, Domains, and Conserved Motifs

**Figure 2:**
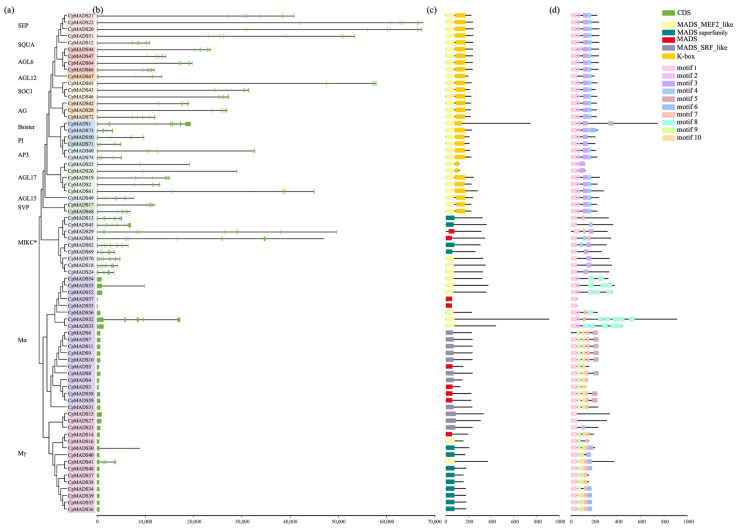
Gene structure, conserved domains, and conserved motifs analysis of *CpMADS*. (**a**) Phylogenetic tree of the *CpMADS* family. (**b**) Intron–exon structure analysis. Green boxes represent exons, while black lines represent introns. (**c**) Conserved domain analysis of *C. praecox* CpMADS proteins. (**d**) Conserved motif analysis. Motifs 1–10 are represented by boxes of different colors, with the length of each box corresponding to motif length.

Alignment of the CDS sequences of C. praecox MADS genes with their genomic DNA revealed considerable variation among genes in sequence length, exon number, and exon length (Figure 2b). The exon count varied between 1 and 12, with CpMADS29 (Cpra05G01340.1) having the maximum number of exons, totaling 12. Among the Type I MADS-box genes, 30 genes contained only one exon, whereas *CpMADS41* (*Cpra06G00606.1*) had the most exons, with seven in total. In the analysis of conserved domains (Figure 2c), all CpMADS proteins contained MADS, MADS_SRF_like, MADS_MEF2_like, and MADS_superfamily domains. The K-box domain was only present in the MIKCC-type members of Type II proteins, further supporting the significant structural differences between Type I and Type II proteins. Additionally, 10 conserved motifs were identified in *C. praecox* MADS proteins (Figure 2d). Motif 1, the core feature of the MADS domain, was present in all proteins except CpMADS6. Among the Type II MADS proteins, motif 3 was present in all except CpMADS13 and CpMADS45. Motifs 3 and 4 were specifically distributed in Type II genes, whereas motifs 6–10 were only detected in Type I genes, indicating clear structural differences between the two types of MADS proteins.

### 2.4. Identification and Characterization of SSR Loci in the CpMADS Gene Family

**Figure 3:**
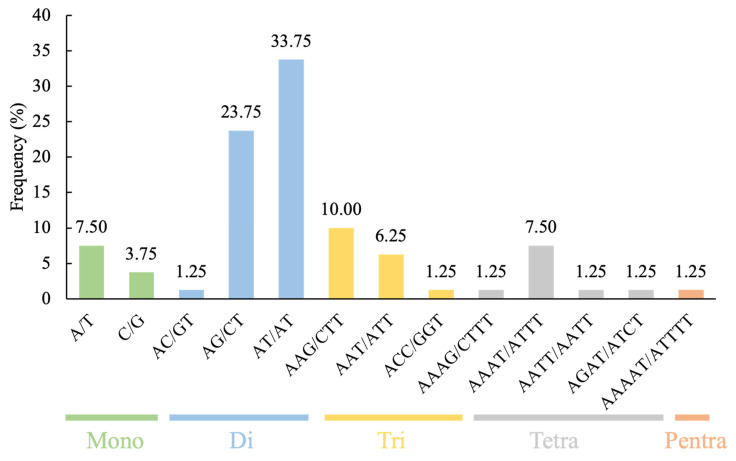
Proportion of different repeat motifs in SSR loci. Green, blue, yellow, gray, and orange distributions represent mononucleotide, dinucleotide, trinucleotide, tetranucleotide, and pentanucleotide repeats, respectively.

Among the 74 *CpMADS* gene sequences (each including 2000 bp of 5′UTR and 3′UTR regions), 41 sequences were found to contain 80 SSR loci. Using Primer3 software, 295 primer pairs were designed (Table 1 and Table S2). These SSRs included five types of repeat motifs: mononucleotide, dinucleotide, trinucleotide, tetranucleotide, and pentanucleotide, with three SSRs in the composite form. Dinucleotide repeats were the predominant type, constituting 58.75% (47) of all SSRs, with trinucleotide repeats next in abundance (14, 17.5%), mononucleotide and tetranucleotide repeats (9, 11.25% each), and pentanucleotide repeats, which were the least common (1, 1.25%) (Table 2). SSR loci with repeat counts > 20 (16.25%) and >6 (15%) were the most abundant (Table 2). Among the dinucleotide repeat sequences, the AT/AT type was the most common (33.75%), followed by the AG/CT type (32.75%). Among the trinucleotide repeat sequences, the AAG/CTT type was the most frequent (10%) (Figure 3).

### 2.5. Chromosomal Localization of CpMADS and SSR Loci

**Figure 4:**
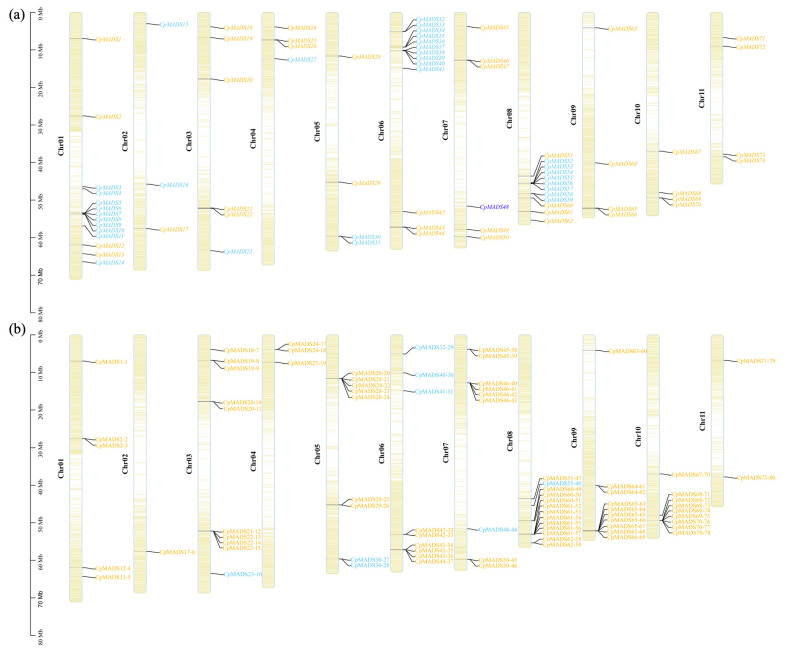
Chromosomal localization of *CpMADS* genes and SSR markers. (**a**) Chromosomal localization of *CpMADS* genes. (**b**) Chromosomal localization of SSR markers. Blue and orange represent CpMADS genes from Type I and Type II, respectively.

To investigate the distribution of *C. praecox* MADS genes and SSR markers on chromosomes, the localization of 74 *CpMADS* genes and SSR markers was analyzed. Using the *C. praecox* genome, these genes were assigned to the physical map of *C. praecox* chromosomes (Figure 4a). The analysis revealed that CpMADS genes are spread across 11 chromosomes, with chromosome 1 containing the most genes (13 in total) and chromosome 3 containing the fewest genes (only 3). The SSR markers were distributed across all chromosomes, with chromosome 8 containing the most SSR markers (13 in total). Further analysis revealed that the majority of SSR markers were located in Type II MADS genes, while Type I MADS genes contained only eight SSR markers, indicating a significant distribution difference of SSR markers among different subfamilies of *CpMADS* genes (Figure 4b; Table S2).

### 2.6. Polymorphism and Universality of SSR Markers

Fifty pairs of SSR primers targeting di- and trinucleotide repeats were randomly chosen for genotyping. The results showed that 43 primer pairs successfully amplified the target DNA fragments, with 10 primer pairs exhibiting polymorphisms in the samples (Figure S1). All polymorphic loci were dinucleotide repeat motifs with repeat numbers ranging from 10 to 25 and fragment lengths ranging from 20 to 55 bp. Genotyping of the 23 samples identified a total of 75 alleles, with each locus exhibiting between 5 and 9 alleles, averaging 7.5 alleles per SSR marker. The number of effective alleles (Ne) per locus ranged from 4.81 on average, with *CpMADS60-50* showing the highest Ne (5.90), and *SSR28* showing the lowest (5.31). The Shannon diversity index (I) varied between 1.38 and 1.94, averaging 1.70, which reflects a high level of genetic diversity among the samples. The expected heterozygosity (He) ranged from 0.71 to 0.83, with an average of 0.79; observed heterozygosity (Ho) ranged from 0.21 to 0.91, with an average of 0.72. The polymorphic information content (PIC) ranged from 0.74 to 0.82, with an average of 0.78, indicating that these SSR markers are suitable for genetic diversity analysis (Table 3).

### 2.7. Unweighted Pair Group Method with Arithmetic (UPGMA) Cluster Analysis of Different Varieties of C. praecox Based on CpMADS Markers

**Figure 5:**
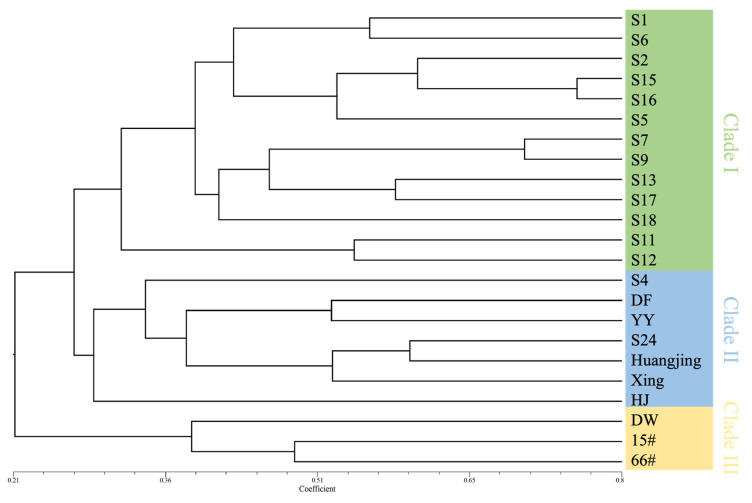
A phylogenetic tree comprising 23 *C. praecox* varieties was generated using UPGMA clustering implemented in NTSYS-pc version 2.0. Comprehensive details of sample collection for these varieties are listed in Table S3.

An UPGMA-based phylogenetic tree was employed to illustrate the genetic relationships among the 23 C. praecox varieties (Figure 5). The analysis revealed that the 23 *C. praecox* varieties were divided into three main clades: the first clade (Clade I) included S1, S6, S2, S15, S16, S5, S7, S9, S13, S17, S18, S11, and S12; the second clade (Clade II) included S4, DF, YY, S24, Huangjin, Xing, and HJ; and the third clade (Clade III) consisted of DW, 15#, and 66#. These results indicate that these varieties are genetically closely related.

## 3. Discussion

The MADS-box gene family plays a key role in various aspects of plant growth and development, especially in controlling flowering time and the formation of floral organs [45]. Wintersweet (*C. praecox*), a traditional Chinese flower, blooms in midwinter from November to February of the following year, with fragrant flowers offering high ornamental value [46]. In recent years, cut flowers have become popular because of their long vase life and strong fragrance [47]. Research on the MADS-box gene family can provide new understanding of genetic improvement and breeding of wintersweet flowers. A total of 74 MADS-box genes were detected through bioinformatics analysis in this study. Based on the classification method used for *A. thaliana*, these genes were divided into two types: Type I (35 genes) and Type II (39 genes). Type I includes 20 Mα subfamily members and 15 Mγ subfamily members, with no Mβ subfamily genes identified, suggesting they may have been lost during evolution. Type II includes 12 subfamilies: *SEP* (4), *SQUA* (1), *AGL6* (4), *AGL12* (1), *SOC1* (3), *AG* (3), sister (2), *PI* (2), *AP3* (2), *AGL17* (5), *AGL15* (1), and *SVP* (2). Interestingly, no *FLC* homologs were found in wintersweet, similar to *Citrus sinensis* (sweet orange), and consistent with previous study [41,48]. So far, the number of identified MADS-box family members includes 107 in *A. thaliana* [5], 106 in *Glycine max* [49], 98 in *Pisum sativum* [12], 131 in *Solanum lycopersicum* [50], and 57 in *Sesamum indicum* L. [51]. The variation in the number of MADS-box genes across species may be attributed to differences in genome size and the degree of whole-genome duplication events. Physicochemical analysis revealed significant variations in protein sequence length, isoelectric point, and hydrophilicity among the family members, which may be related to their specific functions. Gene structure analysis showed that the structure of Type I MADS-box genes in wintersweet is simpler than that of Type II, with most members lacking introns, similar to species such as *Rhododendron griersonianum* Balf. f. et Forrest [52], *Perilla frutescens* [53], *Malus domestica* [54], *Cucumis sativus* [55], and *Citrus* [22]. Motif analysis showed that conserved motifs predominantly occurred within groups and contributed significantly to species-specific roles. All MADS-box genes contain one MADS-conserved domain, whereas Type II members also contain a unique K-box domain, indicating that Type II genes have more complex functions. This is similar to findings in *Arabidopsis* [5], *Solanum melongena* [50], and *Solanum tuberosum* [56]. Evolutionary and conserved domain analyses supported these findings, as genes sharing the same conserved domains tended to cluster together with the phylogenetic tree classification resembling that of *Arabidopsis*, suggesting a relatively conserved evolutionary trend in the MADS-box gene family across species.

In recent years, SSR markers have emerged as a dependable tool for assessing genetic diversity and developing genetic linkage maps, primarily due to their codominant nature and high allelic variation at individual loci. Although many SSR markers have been established for wintersweet, those specifically derived from the MADS-box gene family remain undeveloped [27,57,58]. In this study, SSR loci within the CpMADS gene family were screened using MISA, revealing that dinucleotide repeats were the predominant type (58.75%), with trinucleotide repeats accounting for 17.5% (Table 3, Figure 3). This aligns with the SSR motifs identified in the *CpTPS* gene family of wintersweet, but contrasts with findings from the wintersweet transcriptome, where trinucleotide repeat sequences were the predominant type. The AT/AT type was the most abundant dinucleotide repeat sequence, consistent with the CpTPSMS results [26] but inconsistent with the wintersweet transcriptome results [27]. The AAG/CTT type was the most abundant trinucleotide repeat sequence, which is the same as that found in the wintersweet transcriptome, but inconsistent with the CpTPSMS results [26,27]. These differences could be due to the SSR search parameters, dataset size, and the tools used for database mining [59]. The *CpMADS* genes were distributed across 11 chromosomes, with the greatest number located on chromosome 1, whereas SSRs in *CpMADS* were most abundant on chromosome 8, indicating that the *CpMADS* SSRs were unevenly distributed (Figure 4). Type II *CpMADS* genes outnumber Type I genes, likely because of the greater number and length of introns in Type II *CpMADS* genes. Many of the *ABCDE* genes closely related to flower organ development belong to the Type II *CpMADS* gene family, suggesting that the abundance of these SSR loci is closely related to the diversity in the morphology and number of wintersweet flower organs. Identifying and analyzing these polymorphic SSRs originating from MADS genes will aid in uncovering their functional properties and offer a theoretical foundation for developing functional SSR markers. Markers with a PIC value exceeding 0.5 were regarded as highly informative [60]. In this study, all 10 polymorphic primers exhibited PIC values exceeding 0.5, demonstrating high polymorphism. Cluster analysis grouped the 23 wintersweet varieties into three main clades. Thirteen varieties clustered into Clade I, the largest group, indicating high genetic similarity at the *CpMADS* gene level among these varieties, possibly because of similar genetic backgrounds or closely related breeding materials. Clustering analysis placed seven varieties in Clade II and three in Clade III. However, it should be noted that this clustering was based solely on molecular marker data without incorporating phenotypic trait information. The absence of phenotypic data limits the biological interpretation of the clustering results and may overlook variations relevant to observable characteristics. Future studies integrating both molecular and phenotypic data would provide a more comprehensive understanding of genetic diversity and relationships among *C. praecox* cultivars.

## 4. Materials and Methods

### 4.1. Plant Materials and DNA Extraction

The 23 varieties of wintersweet were cultivated in the wintersweet resource garden at Southwest University (Chongqing, China) and maintained under natural conditions for polymorphism identification (Table S3). Flowers were collected at full bloom. DNA was extracted from plant tissue using the CTAB method [61].

### 4.2. Identification and Characterization of the CpMADS Gene Family

To identify MADS-box genes, the predicted protein sequences from the *C. praecox* genome were first scanned using HMMER v3.0 with hidden Markov model (HMM) profiles PF00319 (SRF-TF domain) and PF01486 (K-box domain) obtained from the Pfam database. The initial candidates were then subjected to domain verification through the NCBI Conserved Domain Database (CDD), and only sequences containing a complete MADS domain were retained as putative CpMADS. The physico-chemical characteristics of the identified CpMADS proteins were analyzed using ExPASy ProtParam (http://web.expasy.org/protparam/; accessed 8 March 2025), while their in-silico subcellular localization was predicted with Cell-PLoc 2.0 (version 2.0; http://www.csbio.sjtu.edu.cn/bioinf/Cell-PLoc-2/, accessed on 8 March 2025). Multiple sequence alignments were performed using MEGA X [62], and a phylogenetic tree was generated with the neighbor-joining (NJ) method supported by 1000 bootstrap replicates.

### 4.3. Gene Structure, Protein Motif, and Conserved Domain Analyses

Intron–exon structures were analyzed and visualized using the Amazing Optional Gene Viewer module and Gene Structure View (Advanced) plugin in TBtools [63]. Conserved domains of MADS proteins were identified through NCBI’s Batch CD-Search tool. Conserved motifs were identified using the MEME Suite online tool (https://meme-suite.org/meme/, accessed 13 March 2025), with the maximum number of motifs specified as 10.

### 4.4. Chromosomal Distribution of MADS-Box Genes and SSR Loci

The chromosomal positions of the MADS-box genes were extracted from the whole-genome annotation data. Chromosome coloring was carried out using the Gene Density Profile plugin in TBtools [63], and the locations of MADS genes along with SSR loci on the wintersweet chromosomes were determined using the Gene Location Visualization tool for GTF/GFF files within TBtools.

### 4.5. Microsatellite Marker Identification, PCR Amplification, and Data Analysis

The *CpMADS* gene sequences (including 2000 bp of 5′UTR and 2000 bp of 3′UTR) were extracted from the genome using the Gtf/Gff3 sequence extraction tool in TBtools [63]. Microsatellite loci were detected using MISA software [64] based on the following criteria: repeat number of mononucleotide repeats ≥ 20, repeat number of dinucleotide repeats ≥ 10, and repeat number of other types of repeats ≥ 5. Primers were designed with Primer3 software [65], and 50 primer pairs generating products between 100 and 300 bp were randomly selected. These primers were used to amplify 23 wintersweet varieties to study SSR locus polymorphisms. PCR products were resolved on an 8% polyacrylamide gel, and SSRs matching the expected length were screened for polymorphic potential. Electrophoresis of PCR products was performed in 1× TBE buffer (200 V, 1 h) with a 2000 bp marker for size reference. Silver staining was used to visualize the bands. In SSR data analysis, band sizes were scored manually, with absent bands marked as “0” and present bands as “1.” The effective allele number (Ne), allele number (Na), Shannon information index (I), and fixation index (F) were calculated for each locus using GenAlEx 6.5 software [66]. The UPGMA clustering analysis was performed using NTSYS-pc 2.0 [67].

## 5. Conclusions

This study identified 74 members of the MADS-box gene family in *C. praecox*. Phylogenetic analysis categorized the genes into Type I and Type II, where Type I was further subdivided into Mα and Mγ subfamilies, and Type II comprised 12 distinct subfamilies. SSR locus screening of *CpMADS* genes led to the identification of 80 SSR markers. A total of 10 pairs of highly polymorphic primers were successfully selected, with an average of seven SSR variations detected per locus. All polymorphic primers had PIC values greater than 0.5, indicating high polymorphism. Cluster analysis grouped the 23 wintersweet varieties into three major clades. These results not only validate the effectiveness of *CpMADS* genes as functional markers in distinguishing wintersweet varieties but also provides clues for further exploration of their role in flower development. It also lays a theoretical foundation for the subsequent use of functional markers in MAS to screen for superior germplasm.

## Figures and Tables

**Table 1:** Results of the microsatellite search on *CpMADS* genes.

Item	Number
Total sequences analyzed	74
Total length of sequences analyzed (bp)	1,209,009
Total SSRs identified	80
Number of sequences containing SSRs	41
Number of sequences with multiple SSRs	21
Number of compound SSRs	3
Number of SSR primers designed	295
Total repeat motifs detected	29

**Table 2:** Relationship between the distribution frequency of different SSR motif types and their repeat numbers in *CpMADS* genes.

Number of Repetitions	Mononucleotide (Mono)	Dinucleotide (Di)	Trinucleotide (Tri)	Tetranucleotide (Tetra)	Pentanucleotide (Pentra)	Total	Percentage (%)
5	0	0	0	5	1	6	7.5
6	0	0	9	3	0	12	15
7	0	0	3	1	0	4	5
8	0	0	0	0	0	0	0
9	0	0	1	0	0	1	1.25
10	0	6	0	0	0	6	7.5
11	0	8	1	0	0	9	11.25
12	0	6	0	0	0	6	7.5
13	0	3	0	0	0	3	3.75
14	0	5	0	0	0	5	6.25
15	0	3	0	0	0	3	3.75
16	0	2	0	0	0	2	2.5
17	0	3	0	0	0	3	3.75
18	0	0	0	0	0	0	0
19	0	1	0	0	0	1	1.25
20	5	1	0	0	0	6	7.5
>20	4	9	0	0	0	13	16.25
Total	9	47	14	9	1	80	
Percentage (%)	11.25	58.75	17.5	11.25	1.25		

**Table 3:** Details regarding the sequences and genetic variation of the 10 SSR markers.

Locus	Forward Primer (5′–3′)	Reverse Primer (5′–3′)	Na	Ne	I	Ho	He	PIC
*CpMADS41-31*	CCGATCAGAGCTGAATCCCC	GGAACGTCCCTGATAACGCA	7	4.60	1.71	0.70	0.78	0.76
*CpMADS60-50*	GGTTTTGAGTTCGATTTCTCCCT	GGTTTTGAGTTCGATTTCTCCCT	9	5.90	1.94	0.45	0.83	0.82
*CpMADS61-53*	CAGTCAAGCCCCAACCTGAT	CCCCCAAACCCACCACTATC	7	5.60	1.80	0.74	0.82	0.80
*CpMADS23-16*	AGCAATATTGGCCATGTGGG	AGCAGAGGTGAAAGTATCCGC	7	5.44	1.78	0.91	0.82	0.81
*CpMADS66-69*	AGGTGAGCACATGTGAGTGA	TTCTCCCGACTTTGGCTGAA	8	4.79	1.76	0.78	0.79	0.76
*CpMADS19-9*	TCACATGTGTATCCTTAAACCGT	TGTCGATTTCAAGTGCATCAAA	8	5.39	1.84	0.21	0.81	0.82
*CpMADS28-20*	CGGGAAAAGCATTTCGACCG	AGTCCGTAATCTCAGGCGAG	6	3.74	1.47	0.81	0.73	0.74
*CpMADS46-41*	TCATTTTCGGGCCTTGTCCA	CAATGCGTGGAAACAGCACA	6	5.31	1.72	0.89	0.81	0.82
*CpMADS50-46*	GCAACAAAACACAAGGCTGG	TGTGTTTGTGAGGGAGGCAA	7	3.87	1.60	0.86	0.74	0.74
*CpMADS51-47*	GCCCTAAGGTTTGCTAGAGGA	AACGGCAACACCCAAATTGG	5	3.50	1.38	0.82	0.71	0.75
Mean			7	4.81	1.70	0.72	0.79	0.78

## Data Availability

Data are contained within the article and Supplementary Materials.

## Supplementary Materials

The following supporting information can be downloaded at https://www.mdpi.com/article/10.3390/plants14152450/s1, Table S1: The detailed information of 74 *CpMADS* genes identified in wintersweet genome; Table S2. SSR loci in *CpMADS* genes; Table S3: Sample collection of 23 *Chimonanthus praecox* varieties. Figure S1. Polymorphism of primers *CpMADS19-9* and *CpMADS60-50* in 23 *Chimonanthus praecox* varieties.
