# Supplementary material for: Identification of the MADS-Box Gene Family and Development of Simple Sequence Repeat Markers in Chimonanthus praecox
Source: Plants (Basel). 2025 Aug 7;14(15):2450. doi: 10.3390/plants14152450 (PMC12349607; doi:10.3390/plants14152450)
Supplement: Supplementary file 1 [file plants-14-02450-s001.zip › plants-3742712-supplementary.pdf]

Table S1 The detailed information of 74 *CpMADS* genes identified in wintersweet genome

**Sequence ID**	**Gene name**	**Number of Amino Acid**	**Molecular Weight**	**Theoretical pI**	**Instability Index**	**Aliphatic Index**	**Grand Average of Hydropathicity**	**In-silico subcellular localisation**
Cpra01G00538.1	CpMADS1	746	82273.42	6.93	53.86	85.54	-0.248	Nucleus
Cpra01G01757.1	CpMADS2	229	26363.01	9.61	48	83.84	-0.662	Nucleus
Cpra01G02123.1	CpMADS3	130	14656.72	6.74	52.24	77.15	-0.598	Nucleus
Cpra01G02133.1	CpMADS4	147	16888.55	9.05	49.41	81.56	-0.601	Nucleus
Cpra01G02299.1	CpMADS5	155	17420.81	4.92	58.61	88.06	-0.55	Nucleus
Cpra01G02300.1	CpMADS6	229	25692.86	4.56	61.25	81.31	-0.463	Nucleus
Cpra01G02303.1	CpMADS7	236	26371.74	4.49	60.18	85.51	-0.381	Nucleus
Cpra01G02314.1	CpMADS8	237	26595.96	4.69	50.25	72.03	-0.477	Nucleus
Cpra01G02320.1	CpMADS9	236	26501.83	4.5	56.1	83.05	-0.436	Nucleus
Cpra01G02322.1	CpMADS10	236	26450.79	4.58	57.47	82.2	-0.465	Nucleus
Cpra01G02425.1	CpMADS11	236	26619.97	4.63	60.05	82.2	-0.475	Nucleus
Cpra01G02706.1	CpMADS12	242	27964.98	9.1	55.29	81.45	-0.733	Nucleus
Cpra01G02888.1	CpMADS13	324	37159.89	6.66	41.49	95.96	-0.324	Nucleus
Cpra01G03031.1	CpMADS14	199	21979.57	4.82	60.98	82.36	-0.532	Nucleus
Cpra02G00239.1	CpMADS15	334	38866.48	8.11	61.33	63.29	-0.683	Nucleus
Cpra02G01105.1	CpMADS16	156	17675.09	5.97	63.21	93.14	-0.377	Nucleus
Cpra02G01509.1	CpMADS17	225	25222.84	5.42	59.37	88.84	-0.493	Nucleus
Cpra03G00302.1	CpMADS18	352	39623.38	6.68	39.24	78.12	-0.639	Nucleus
Cpra03G00536.1	CpMADS19	247	28594.87	9.15	49.76	78.18	-0.686	Nucleus
Cpra03G01158.1	CpMADS20	245	28135.07	8.62	47.4	77.18	-0.615	Nucleus
Cpra03G01640.1	CpMADS21	226	25915.59	9.22	57.53	77.74	-0.715	Nucleus
Cpra03G01641.1	CpMADS22	241	27468.17	9.11	44.82	81.37	-0.673	Nucleus
Cpra03G02328.1	CpMADS23	236	27043.38	9.16	49.47	75.59	-0.534	Nucleus
Cpra04G00311.1	CpMADS24	328	36816.61	7.1	46.81	72.59	-0.579	Nucleus
Cpra04G00579.1	CpMADS25	120	13444.62	9.79	30.2	94.92	-0.206	Nucleus
Cpra04G00582.1	CpMADS26	125	14556.67	9.69	43.98	81.04	-0.571	Nucleus
Cpra04G00909.1	CpMADS27	308	35664.69	6.6	62.01	65.84	-0.66	Nucleus
Cpra05G00791.1	CpMADS28	222	25637.96	9.17	48.82	77.84	-0.757	Nucleus
Cpra05G01340.1	CpMADS29	315	35948.06	8.86	65.49	83.27	-0.591	Nucleus
Cpra05G02184.1	CpMADS30	207	23305.49	5.43	63.63	81.06	-0.568	Nucleus
Cpra05G02185.1	CpMADS31	234	26225.43	4.77	66.35	77.18	-0.456	Nucleus
Cpra06G00287.1	CpMADS32	912	99621.54	4.8	46.62	80.71	-0.221	Nucleus
Cpra06G00288.1	CpMADS33	443	48346.07	4.41	50.1	74.81	-0.342	Nucleus
Cpra06G00472.1	CpMADS34	177	20237.24	7.82	50.17	82.09	-0.553	Nucleus
Cpra06G00474.1	CpMADS35	181	20361.29	6.97	63.59	87.85	-0.455	Nucleus
Cpra06G00475.1	CpMADS36	181	20409.38	7.77	64.01	87.85	-0.443	Nucleus
Cpra06G00510.1	CpMADS37	157	17822.42	8.95	59.29	86.31	-0.563	Nucleus
Cpra06G00512.1	CpMADS38	157	17825.43	8.63	59.29	86.31	-0.526	Nucleus
Cpra06G00517.1	CpMADS39	180	20503.5	8.47	54.8	85.61	-0.513	Nucleus

Cpra06G00518.1	CpMADS40	171	19556.45	7.02	51.16	81.46	-0.534	Nucleus
Cpra06G00606.1	CpMADS41	372	41925.61	5.37	44.62	81.75	-0.48	Nucleus
Cpra06G01706.1	CpMADS42	223	25747.29	9.17	63.86	79.19	-0.711	Nucleus
Cpra06G02035.1	CpMADS43	214	24783.34	9.4	55.98	87.06	-0.746	Nucleus
Cpra06G02036.1	CpMADS44	241	27699.56	9.37	52.97	80.91	-0.712	Nucleus
Cpra07G00272.1	CpMADS45	362	41808.29	7.78	51.32	70.06	-0.694	Nucleus
Cpra07G00939.1	CpMADS46	225	25990.64	9.18	63.88	83.64	-0.798	Nucleus
Cpra07G00941.1	CpMADS47	240	27405.01	8.94	48.73	79.25	-0.75	Nucleus
Cpra07G01628.1	CpMADS48	181	20744.67	5.98	57.84	84.03	-0.557	Nucleus
Cpra07G01892.1	CpMADS49	240	27489.83	8.13	50.48	94.29	-0.477	Nucleus
Cpra07G01982.1	CpMADS50	211	24600.25	9.5	46.77	81.33	-0.836	Nucleus
Cpra08G01315.1	CpMADS51	243	28435.27	8.68	49.21	80.29	-0.806	Nucleus
Cpra08G01358.1	CpMADS52	359	39200.95	4.2	64.87	73.87	-0.277	Nucleus
Cpra08G01360.1	CpMADS53	54	6059.17	10.29	88.41	85	-0.304	Nucleus
Cpra08G01368.1	CpMADS54	322	35216.55	4.34	63.45	75.4	-0.327	Nucleus
Cpra08G01369.1	CpMADS55	376	41254.42	4.4	60.27	73.14	-0.355	Nucleus
Cpra08G01370.1	CpMADS56	231	24921.07	4.96	62.6	76.84	-0.341	Nucleus
Cpra08G01373.1	CpMADS57	58	6451.67	10.29	86.33	87.59	-0.24	Nucleus
Cpra08G01442.1	CpMADS58	225	24891.11	4.97	67.42	70.58	-0.46	Nucleus
Cpra08G01444.1	CpMADS59	225	24876.16	4.89	67.86	72.76	-0.389	Nucleus
Cpra08G01485.1	CpMADS60	215	24864.93	9.15	43.39	79.35	-0.839	Nucleus
Cpra08G01638.1	CpMADS61	281	32216.96	9.49	53.4	83.2	-0.621	Nucleus
Cpra08G01800.1	CpMADS62	306	34212.94	4.35	52.2	80.56	-0.558	Nucleus
Cpra09G00198.1	CpMADS63	346	39344.38	5.63	60.6	78.93	-0.684	Nucleus
Cpra09G01076.1	CpMADS64	238	26997.72	8.16	42.06	75	-0.664	Nucleus
Cpra09G02014.1	CpMADS65	228	26479.28	9.23	54.93	77.85	-0.832	Nucleus
Cpra09G02015.1	CpMADS66	237	27153.94	8.78	41.98	74.51	-0.71	Nucleus
Cpra10G00953.1	CpMADS67	200	22708.36	6.24	56.87	87.8	-0.321	Nucleus
Cpra10G01515.1	CpMADS68	226	25696.37	5.91	44.2	79.38	-0.703	Nucleus
Cpra10G01621.1	CpMADS69	264	29527.9	4.95	67.76	69.43	-0.698	Nucleus
Cpra10G01622.1	CpMADS70	330	37410.24	5.97	58	74.45	-0.582	Nucleus
Cpra11G00433.1	CpMADS71	209	24259.76	7.78	58.47	86.32	-0.789	Nucleus
Cpra11G00558.1	CpMADS72	221	25442.98	9.25	52.9	87.87	-0.622	Nucleus
Cpra11G01038.1	CpMADS73	232	27129.93	7.76	64.83	79.87	-0.685	Nucleus
Cpra11G01073.1	CpMADS74	226	25819.95	8.81	42.36	72.08	-0.865	Nucleus

Table S2. SSR loci in *CpMADS* genes

**ID**	**Site name**	**SSR**	**ID**	**Site name**	**SSR**
Cpra01G00538	CpMADS1-1	(TA)31	Cpra07G00939	CpMADS46-41	(TC)11
Cpra01G01757	CpMADS2-2	(AG)10	Cpra07G00939	CpMADS46-42	(CA)11
Cpra01G01757	CpMADS2-3	(AT)12	Cpra07G00939	CpMADS46-43	(TA)11
Cpra01G02706	CpMADS12-4	(TA)10	Cpra07G01628	CpMADS48-44	(GA)20
Cpra01G02888	CpMADS13-5	(CTT)6	Cpra07G01982	CpMADS50-45	(TTC)7

Cpra02G01509	CpMADS17-6	(AT)23	Cpra07G01982	CpMADS50-46	(TC)11
Cpra03G00302	CpMADS18-7	(TA)15	Cpra08G01315	CpMADS51-47	(AT)15
Cpra03G00536	CpMADS19-8	(TCT)6	Cpra08G01369	CpMADS55-48	(G)30
Cpra03G00536	CpMADS19-9	(AT)11	Cpra08G01485	CpMADS60-49	(A)20
Cpra03G01158	CpMADS20-10	(AAT)6	Cpra08G01485	CpMADS60-50	(AT)25
Cpra03G01158	CpMADS20-11	(TTTAT)5	Cpra08G01485	CpMADS60-51	(AT)10
Cpra03G01640	CpMADS21-12	(TCT)6	Cpra08G01638	CpMADS61-52	(A)20
Cpra03G01641	CpMADS22-13	(TA)12	Cpra08G01638	CpMADS61-53	(AG)17
Cpra03G01641	CpMADS22-14	(ATAA)5	Cpra08G01638	CpMADS61-54	(T)20
Cpra03G01641	CpMADS22-15	(AT)19	Cpra08G01638	CpMADS61-55	(GA)14
Cpra03G02328	CpMADS23-16	(AT)10	Cpra08G01638	CpMADS61-56	(TAT)11
Cpra04G00311	CpMADS24-17	(TC)29	Cpra08G01638	CpMADS61-57	(AT)14
Cpra04G00311	CpMADS24-18	(TCTA)6*	Cpra08G01800	CpMADS62-58	(T)20
Cpra04G00579	CpMADS25-19	(AT)13	Cpra08G01800	CpMADS62-59	(GGT)7
Cpra05G00791	CpMADS28-20	(TC)14	Cpra09G00198	CpMADS63-60	(G)21
Cpra05G00791	CpMADS28-21	(TC)16	Cpra09G01076	CpMADS64-61	(ATT)9
Cpra05G00791	CpMADS28-22	(A)20	Cpra09G01076	CpMADS64-62	(TTTC)5
Cpra05G00791	CpMADS28-23	(TCT)6	Cpra09G02014	CpMADS65-63	(AGA)7
Cpra05G00791	CpMADS28-24	(AAAT)5	Cpra09G02014	CpMADS65-64	(TA)11
Cpra05G01340	CpMADS29-25	(TC)15	Cpra09G02014	CpMADS65-65	(TA)27
Cpra05G01340	CpMADS29-26	(TA)10	Cpra09G02014	CpMADS65-66	(AT)21
Cpra05G02184	CpMADS30-27	(AT)13	Cpra09G02014	CpMADS65-67	(TTTA)6
Cpra05G02184	CpMADS30-28	(AGA)6	Cpra09G02014	CpMADS65-68	(TTTA)7
Cpra06G00287	CpMADS32-29	(TC)13	Cpra09G02015	CpMADS66-69	(AG)21
Cpra06G00518	CpMADS40-30	(TA)14	Cpra10G00953	CpMADS67-70	(CT)25
Cpra06G00606	CpMADS41-31	(AG)25	Cpra10G01515	CpMADS68-71	(AG)12
Cpra06G01706	CpMADS42-32	(TTAT)5	Cpra10G01515	CpMADS68-72	(CT)10
Cpra06G01706	CpMADS42-33	(AT)14	Cpra10G01515	CpMADS68-73	(A)22
Cpra06G02035	CpMADS43-34	(C)27	Cpra10G01621	CpMADS69-74	(TA)12
Cpra06G02035	CpMADS43-35	(AT)11	Cpra10G01621	CpMADS69-75	(CT)11
Cpra06G02035	CpMADS43-36	(CT)17	Cpra10G01622	CpMADS70-76	(CTT)6
Cpra06G02036	CpMADS44-37	(TTA)6	Cpra10G01622	CpMADS70-77	(AATA)6
Cpra07G00272	CpMADS45-38	(TA)16	Cpra10G01622	CpMADS70-78	(TA)12
Cpra07G00272	CpMADS45-39	(TTAA)5	Cpra11G00433	CpMADS71-79	(CT)17
Cpra07G00939	CpMADS46-40	(ATT)6	Cpra11G01038	CpMADS73-80	(TA)12

Table S3 Sample collection of 23 *Chimonanthus praecox* varieties

**Individuals**	**Species name**	**Voucher No.**	**Collection location**	**Longitude and latitude**	**Material class**
S1	*C.praecox*	2024_S1	Beibei District, Chongqing City	N 29.766683 E 106.374561	cultivated
S2	*C.praecox*	2024_S2	Beibei District, Chongqing City	N 29.766683 E 106.374561	cultivated
S4	*C.praecox*	2024_S4	Beibei District, Chongqing City	N 29.766683 E 106.374561	cultivated
S5	*C.praecox*	2024_S5	Beibei District, Chongqing City	N 29.766683 E 106.374561	cultivated

S6	*C.praecox*	2024_S6	Beibei District, Chongqing City	N 29.766683 E 106.374561	cultivated
S7	*C.praecox*	2024_S7	Beibei District, Chongqing City	N 29.766683 E 106.374561	cultivated
S9	*C.praecox*	2024_S9	Beibei District, Chongqing City	N 29.766683 E 106.374561	cultivated
S11	*C.praecox*	2024_S11	Beibei District, Chongqing City	N 29.766683 E 106.374561	cultivated
S12	*C.praecox*	2024_S12	Beibei District, Chongqing City	N 29.766683 E 106.374561	cultivated
S13	*C.praecox*	2024_S13	Beibei District, Chongqing City	N 29.766683 E 106.374561	cultivated
S15	*C.praecox*	2024_S15	Beibei District, Chongqing City	N 29.766683 E 106.374561	cultivated
S16	*C.praecox*	2024_S16	Beibei District, Chongqing City	N 29.766683 E 106.374561	cultivated
S17	*C.praecox*	2024_S17	Beibei District, Chongqing City	N 29.766683 E 106.374561	cultivated
S18	*C.praecox*	2024_S18	Beibei District, Chongqing City	N 29.766683 E 106.374561	cultivated
S24	*C.praecox*	2024_S24	Beibei District, Chongqing City	N 29.766683 E 106.374561	cultivated
DF	*C.praecox*	2024_DF	Beibei District, Chongqing City	N 29.766683 E 106.374561	cultivated
DW	*C.praecox*	2024_DW	Beibei District, Chongqing City	N 29.766683 E 106.374561	cultivated
YY	*C.praecox*	2024_YY	Beibei District, Chongqing City	N 29.766683 E 106.374561	cultivated
Xing	*C.praecox*	2024_Xing	Beibei District, Chongqing City	N 29.766683 E 106.374561	cultivated
15#	*C.praecox*	2024_15#	Beibei District, Chongqing City	N 29.766683 E 106.374561	cultivated
66#	*C.praecox*	2024_66#	Beibei District, Chongqing City	N 29.766683 E 106.374561	cultivated
HJ	*C.praecox*	2024_HJ	Beibei District, Chongqing City	N 29.766683 E 106.374561	cultivated
Huangjin	*C.praecox*	2024_Huangjin	Beibei District, Chongqing City	N 29.766683 E 106.374561	cultivated

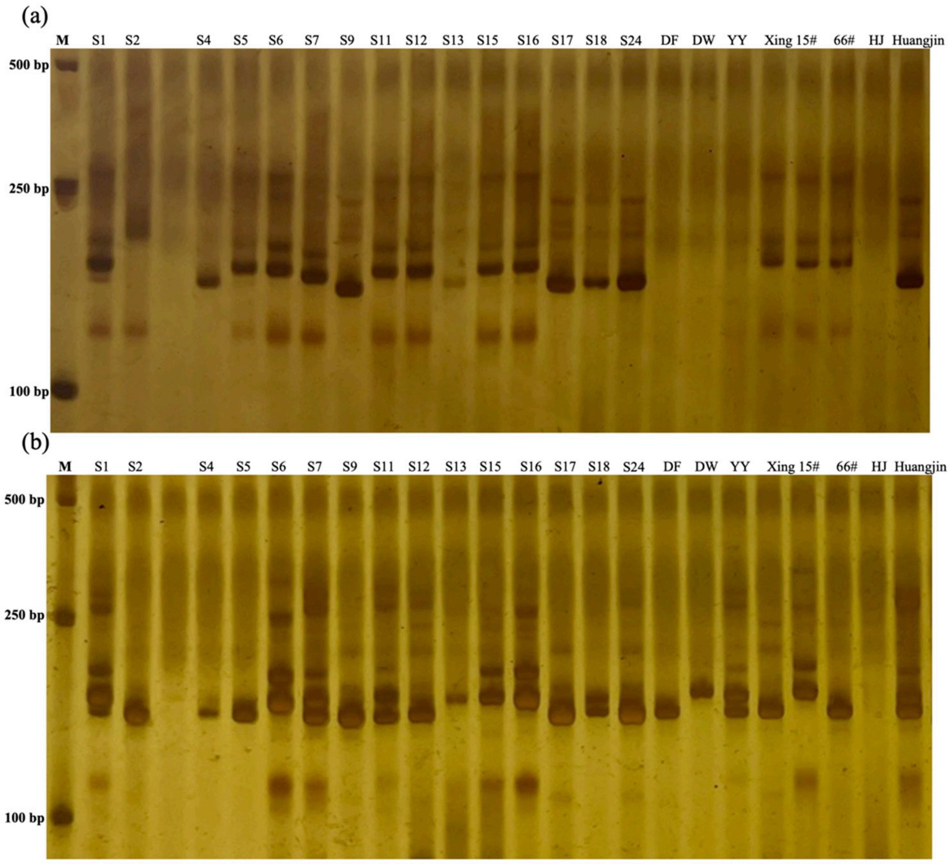

Figure S1. Polymorphism of primers *CpMADS19-9* and *CpMADS60-50* in 23 *Chimonanthus praecox* varieties.
